# Home and Office Blood Pressure Control among Treated Hypertensive Patients in Japan: Findings from the Japan Home *versus* Office Blood Pressure Measurement Evaluation (J-HOME) Study

**DOI:** 10.3390/ph3020419

**Published:** 2010-02-04

**Authors:** Taku Obara, Takayoshi Ohkubo, Michihiro Satoh, Nariyasu Mano, Yutaka Imai

**Affiliations:** 1 Department of Clinical Pharmacology and Therapeutics, Tohoku University Graduate School of Pharmaceutical Sciences and Medicine, 2-1 Seiryo-cho, Aoba-ku, Sendai 980-8575, Japan; E-Mails: dontaku@mail.tains.tohoku.ac.jp (T.O.); sato-m@m.tains.tohoku.ac.jp (M.S.); rinsyo@mail.pharm.tohoku.ac.jp (Y.I.); 2 Department of Pharmacy, Tohoku University Hospital, 1-1 Seiryo-cho, Aoba-ku, Sendai 980-8574, Japan; E-Mail: n-mano@mail.tains.tohoku.ac.jp (N.M.); 3 Department of Planning for Drug Development and Clinical Evaluation, Tohoku University Graduate School of Pharmaceutical Sciences, 6-3 Aoba, Aramaki, Aoba-ku, Sendai 980-8578, Japan

**Keywords:** hypertension, antihypertensive treatment, home blood pressure

## Abstract

Appropriate control of blood pressure (BP) is essential for prevention of future cardiovascular events. However, BP control among treated hypertensive patients has been insufficient. Recently, the usefulness of self-measured BP at home (home BP measurement) for the management of hypertension has been reported in many studies. We evaluated BP control both at home and in the office among treated hypertensive patients in primary care settings in Japan (the J-HOME study). We found poor control of home and office BPs and clarified some factors affecting control. We also examined factors associated with the magnitude of the white-coat effect, the morning–evening BP difference, and home heart rate in this J-HOME study.

## 1. Introduction

A meta-analysis of data from 61 prospective observational studies revealed that cardiovascular morbidity and mortality risks increased with blood pressure (BP) levels in all age groups [1]. Hypertension is more strongly associated with stroke than ischemic heart disease or myocardial infarction in Japan [2]. In the Ohasama study, an epidemiological survey of hypertension using self-measured BP at home (home BP) conducted since 1985 in a general population of Ohasama, located in the northern part of Japan [3,4], home BP was more closely associated with a higher risk of cardiovascular mortality and stroke morbidity than conventional BP. These associations were observed when the initial home BP values (one measurement) were used for the analysis [4]. Since home BP values are obtained under stable conditions, home BP measurements can eliminate the whit-coat effect, and they are highly reproducible and appropriate for the management of hypertensive patients receiving antihypertensive drugs [5].

We conducted the Japan Home versus Office Blood Pressure Measurement Evaluation (J-HOME) study to clarify BP control based on home BP measurement among essential hypertensive patients receiving antihypertensive drugs in primary care settings in Japan [6,7]. We also examined factors affecting control of home and office BPs and several parameters, such as the magnitude of the white-coat effect, the morning–evening BP difference, and the home heart rate (HR). This review summarizes the major findings of the J-HOME study.

## 2. Home BP Measurements

As specified by the Japanese guidelines for home BP measurements, patients were asked to measure their BP once every morning in the sitting position, within 1 h of waking, after more than 2 min of rest, but before drug ingestion and breakfast, and once every evening just before bedtime. They were asked to record the results over a 2-week period [8]. The patients used electronic arm-cuff devices that operate on the basis of the cuff-oscillometric method. All such devices available in Japan have been validated and approved by the Ministry of Health, Labour, and Welfare, Japan [9]. The manufacturers of these devices were Omron Healthcare Co., Ltd., (Kyoto, Japan), A&D Co., Ltd., (Tokyo, Japan), Terumo Co., Ltd., (Tokyo, Japan), and Matsushita Electric Works, Ltd., (Osaka, Japan). The actual model of each device was not provided by the doctors who were involved in the study. All devices for the self-measurement of BP used in the present study were certified as having been adjusted to the Association for the Advancement of Medical Instrumentation (AAMI) standard [9]. However, we were not informed of the calibration and maintenance schedules of these devices; this information was outside the scope of the J-HOME study, which was a survey done to evaluate, on the basis of home BP measurements, the actual BP control that was achieved with antihypertensive treatment in the primary care setting in Japan. The mean of all measurements recorded over the 2-week period was calculated for each patient and used for the analysis. We defined the threshold of controlled home BP as a systolic BP < 135 mmHg and a diastolic BP < 85 mmHg, according to several guidelines [10,11].

## 3. Office BP Measurements

Office BP was measured twice consecutively in the sitting position after a rest of at least 2 min at each regularly scheduled visit by a physician (81.0%) or a nurse (19.0%). The physicians or nurses used either the auscultatory method with a mercury (75.1%) or an aneroid sphygmomanometer (3.3%), or the cuff-oscillometric method with an electronic arm-cuff device (21.6%) that had been validated and approved by the Ministry of Health, Labour, and Welfare, Japan. All automatic devices used in the present study were certified as having been adjusted to the AAMI standard [9]. The office BP value for each patient that was used for the analysis was defined as the average of four measurements taken at two office visits during the time period that home measurements were being taken. The threshold of controlled office BP was defined as a systolic BP < 140 mmHg and a diastolic BP < 90 mmHg, according to several guidelines [10,11].

## 4. Data Collection

Patient information was collected using a questionnaire administered by the attending physicians. Therefore, identification of complications was based on the attending physician’s judgment. Dihydropyridine calcium-channel blockers were classified into two groups: “amlodipine” and “dihydropyridines other than amlodipine”. Amlodipine was the most frequently prescribed medication in the J-HOME study; amlodipine is the most long-acting of the calcium-channel blockers. Therefore, we discriminated between amlodipine and the dihydropyridine calcium-channel blockers other than amlodipine. The term “dihydropyridines other than amlodipine” in the present study, therefore, indicates “dihydropyridine calcium-channel blockers other than amlodipine”. Dihydropyridine calcium-channel blockers other than amlodipine included aranidipine, efonidipine, cilnidipine, nicardipine, nisoldipine, nitrendipine, nifedipine, nilvadipine, barnidipine, felodipine, benidipine, and manidipine.

## 5. Study Population

In March 2003, 7,354 physicians, randomly selected from all of Japan, were invited to take part in this project. Of the 1,477 who agreed to participate, 751 collected data for the study. We asked each doctor to enroll five patients. Physicians could enroll patients according to the following inclusion criteria: (1) informed consent to participate in this study was given; (2) receiving antihypertensive drugs; (3) data on morning home and office BP values and patient’s characteristics were available; and (4) essential hypertension. Therefore, as long as these inclusion criteria were fulfilled, physicians could enroll their patients in this study regardless of patients’ sex, age, and BP levels. The study protocol was approved by the Institutional Review Board of Tohoku University School of Medicine.

Most doctors (79.3%) enrolled five or fewer patients (mean 4.7, median 5, mode 5, range 1-25). By the end of August 2003, 3,586 patients were enrolled. Of these, 66 were excluded because antihypertensive drugs were not prescribed. An additional 120 patients were excluded because insufficient data on morning home and office BP values or patient characteristics were provided. Thus, the study population mainly consisted of 3400 patients (Table 1 and Table 2).

**Table 1:** Characteristics of study subjects.

Age (years)	66.2±10.5
Women (%)	55.2
Body mass index (kg/m^2^)	23.8±3.3
Current smoker (%)	14.2
Current drinker (%)	34.8
History of cerebrovascular disease (%)	16.7
History of ischemic heart disease (%)	8.2
Diabetes mellitus (%)	13.7
Renal disease (%)	5.1
Dyslipidemia (%)	40.2
High uric acid (%)	11.5
Morning home systolic BP (mmHg)	139.6±13.8
diastolic BP (mmHg)	81.7±9.6
heart rate (bpm)	67.2±9.1
Evening home systolic BP (mmHg)	133.7±13.4
diastolic BP (mmHg)	76.9±9.2
heart rate (bpm)	69.6±9.2
Office systolic BP (mmHg)	142.8±14.4
diastolic BP (mmHg)	80.7±9.4

BP, blood pressure; Values are shown as mean ± SD for continuous variables.

**Table 2:** Antihypertensive treatment.

Duration of treatment (months)	29.6±42.8
Number of drugs, mean (n)	1.7±0.9
1 (%)	48.7
2 (%)	35.4
3 (%)	12.3
4 or more (%)	3.6
Class of drugs (including combination therapy) (%)	
Calcium-channel blockers	69.6
Amlodipine	38.9
Dihydropyridines other than amlodipine	30.2
Non_ dihydropyridines	1.6
Angiotensin II receptor blockers	43.6
Angiotensin converting-enzyme inhibitors	16.7
Diuretics	9.3
Alpha-blockers	13.4
Beta-blockers	11.7
Alpha/beta-blockers	3.8

Values are shown as mean ± SD for continuous variables.

## 6. Major Findings of the J-HOME Study

### 6.1. BP control [6,7,12]

Morning BP, evening BP, and office BP were properly controlled in 34%, 53%, and 42%, respectively. The proportion of patients with properly controlled BPs decreased with increasing age. 

Treated masked and white-coat hypertension.

#### 6.1.1. Prevalence of treated masked and white-coat hypertension [7,13,14]

The proportion of treated masked hypertension (controlled office BP and uncontrolled home BP) was 23%, 15%, and 19% on the basis of morning home BP, evening home BP, and the average of morning and evening home BP, respectively. The proportion of treated white-coat hypertension (uncontrolled office BP and controlled home BP) was 14%, 26%, and 19% based on morning home BP, evening home BP, and the average of morning and evening home BP, respectively.

#### 6.1.2. Factors affecting treated masked and white-coat hypertension [7]

Overweight (body mass index ≥25 kg/m^2^), relatively higher office systolic BP level (≥130 mmHg), habitual drinking, and a greater number of prescribed drugs (≥2 drugs) were factors for treated masked hypertension on the basis of morning home BP. Female sex, lower body mass index (<25 kg/m^2^), and relatively lower office systolic BP level (<150 mmHg) were factors for treated white-coat hypertension based on morning home BP.

### 6.2. BP difference

#### 6.2.1. Office-morning BP difference [15]

The mean office-morning systolic/diastolic BP difference was 3.2±16.1/-0.9±9.6 mmHg. Older age (≥65 years), overweight, habitual drinking, a family history of cerebrovascular disease, a history of ischemic heart disease, the use of dihydropyridines other than amlodipine, and the use of alpha-blockers were negatively associated with the magnitude of the office-morning systolic BP difference. The use of amlodipine was positively associated with the magnitude of the office-morning systolic BP difference.

#### 6.2.2. Morning-evening home systolic BP difference [16]

The mean morning-evening systolic/diastolic BP difference was 6.1±10.8/4.8±6.5 mmHg. Uncontrolled morning systolic BP, controlled evening systolic BP, older age, measurement of evening home BP after drinking alcohol, and measurement of evening home BP after bathing were positively associated with the magnitude of the morning-evening systolic BP difference.

### 6.3. Home HR

#### 6.3.1. Home HR control [17,18]

Home HR <70 bpm was defined as normal home HR based on our previous study that demonstrated that patients with home HR ≥70 bpm had a significantly higher risk of cardiovascular disease mortality than those with home HR <70 bpm [17]. The proportion of patients with normal morning home HR and those with normal evening home HR were 64% and 53%, respectively. The proportion of patients with normal home HR with controlled home BP, those with high home HR and controlled home BP, those with normal home HR and uncontrolled home BP, and those with high home HR and uncontrolled home BP were 22.7%, 11.3%, 41.6%, and 24.4% based on morning home BP and HR and 29.8%, 24.2%, 23.5%, and 22.5% based on evening home BP and HR, respectively [18].

#### 6.3.2. Factors affecting home HR [19]

Morning home HR was negatively associated with age and use of beta-blockers and positively associated with habitual smoking, morning home diastolic BP, and presence of diabetes mellitus. Although evening home HR was also associated with variables similar to those for morning home HR, the use of angiotensin converting-enzyme inhibitors was negatively associated only with evening home HR. Morning and evening home HR were significantly associated with age, smoking, or alcohol consumption only in men. 

Overweight, habitual smoking, a lower beta-blocker (including alpha/beta-blockers) prescription rate, and a higher alpha-blocker prescription rate were factors that were associated with high home HR and uncontrolled home BP.

### 6.4. Complications

#### 6.4.1. Diabetes mellitus [20]

The proportions of patients with morning home BP < 135/85 mmHg and those with office BP <140/90 mmHg were similar in diabetic patients (30% and 36%) to those in non-diabetic patients (34% and 43%). In diabetic patients, the proportions of patients with morning home BP < 130/80 mmHg and those with office BP < 130/80 mmHg were only 18% and 12%, respectively. Calcium-channel blockers, angiotensin converting-enzyme inhibitors, and alpha-blockers were more frequently prescribed in diabetic patients than in non-diabetic patients. The average number of drugs prescribed was higher in diabetic patients than in non-diabetic patients. 

#### 6.4.2. Resistant hypertension [21]

The proportion of home resistant hypertension (home systolic BP ≥ 135 mmHg and/or home diastolic BP ≥ 85 mmHg) was 66% in 528 patients with three or more antihypertensive drugs. Older age, office resistant hypertension (office systolic BP ≥ 140 mmHg and/or office diastolic BP ≥ 90 mmHg), history of ischemic heart disease and renal disease, taking four or more antihypertensive drugs, and a lower prescription rate of potassium-sparing diuretics were factors associated with home resistant hypertension.

### 6.5. Prescription of diuretics [22]

Of the 3400 subjects studied, 315 (9.3%) were prescribed diuretics. Patients prescribed diuretics were more likely to be obese and had more complications such as renal disease, dyslipidemia, and high uric acid than those without diuretics. In the majority (95%) of patients prescribed diuretics, combination therapy was used. The most commonly prescribed diuretic was trichlormethiazide (44%), followed by indapamide (15%) and spironolactone (14%). Relatively low dosages of diuretics were generally used.

### 6.6. Effect of previous home BP measurement [23]

The proportion of patients who had taken home BP measurements at the time of recruitment into the J-HOME study was 77%. Taking home BP measurements was associated with older age, male sex, a family history of hypertension, a greater number of antihypertensive drugs, the use of alpha-blockers, and taking antihypertensive drugs in the evening. Home and office BPs were controlled better among patients who had taken home BP measurements previously (morning BP, 36%; evening BP, 56%; and office BP. 44%) than among those who had not (25%, 46%, and 38%, respectively).

## 7. Conclusions

In the J-HOME study, we verified the control of BP based on home BP measurement and the use of antihypertensive drugs among essential hypertensive patients receiving antihypertensive medications in primary care settings in Japan.

In the present study, morning home BP was much higher than evening home BP by 5.9/4.8 mmHg (systolic/diastolic). In other Japanese studies, similar results were observed [24,25,26]. On the other hand, evening home BP values in European studies were generally similar to morning home BP values [27,28]. Evening home BP measurements were obtained under different circumstances in European and Japanese studies. In Europe, evening home BP was generally measured in the early evening (1800-2100 h) [29,30]. In Japan, the Japanese guidelines for home BP measurement recommend that evening home BP be measured just before going to bed [8]. Most Japanese habitually drink alcohol or bathe before going to bed; thus, in Japan, evening home BP was measured after drinking or taking a bath. It has been shown that BP values obtained after drinking or taking a bath are lower than BP values obtained before drinking or taking a bath [31,32,33]. In the J-HOME study, we also reported that measurement of evening BP after drinking and measurement of evening BP after bathing were strongly associated with an increased morning-evening home systolic BP difference [16]. Furthermore, this discrepancy might be due to inappropriate drug administration over the 24-hour period and would call for a better distribution of drugs and/or doses over the 24–hour period [34]. Similarly, in the J-HOME study, morning home HR was lower than evening home HR by 2.4 bpm. This discrepancy might also be affected by the evening home BP measuring conditions (before or after drinking alcohol and bathing). Additionally, this might be attributed at least partially to beta-blockers with a short duration of action being taken in the morning, since beta-blockers were taken in the morning by more than 80% of patients taking beta-blockers in the J-HOME study. However, the net effects of drug treatment on the morning and evening home BP and HR cannot be demonstrated in the J-HOME study, since the present study was a cross-sectional design.

In the Japanese guidelines published in 2009, the Japanese Society of Hypertension (JSH) recommended that evening home BP should be measured before dinner, taking drugs, drinking alcohol, and bathing in order to evaluate the effects of antihypertensive drugs [10], However, in hypertension management, home BP measurement plays a role as a tool not only for the evaluation of drug effects but also for the diagnosis of white-coat hypertension and masked hypertension, as well as the improvement of patients’ drug adherence [35,36]. The JSH also stated that subjects should record conditions of drinking alcohol and/or bathing in addition to the BP values, since prohibition of alcohol consumption and/or bathing before evening BP measurements can lower compliance for home BP measurement itself [10]. Therefore, in Japan, evaluation of morning home BP should take priority over the evaluation of evening home BP, since patients’ high BP values might be underestimated when evaluating only evening home BP.

The proportions of masked hypertension and white-coat hypertension have been reported widely [13,14]. Their proportions were very different because of the difference in conditions of antihypertensive treatment, different types of out-of-office BP measurement (home BP measurement/ambulatory BP measurement), differences in cut-off criteria of out-of-office and office BPs, and differences in conditions of BP measurements (especially evening home BP measurement). However, most prospective studies have demonstrated that, regardless of the conditions mentioned above, masked hypertension is associated with increased cardiovascular risk compared to sustained normotension [37,38,39]. On the other hand, it has been demonstrated that the risk of developing sustained hypertension is higher in subjects with white-coat hypertension than in sustained normotensives [40,41], although the cardiovascular risk of white-coat hypertension is similar to that of sustained normotension [37,38,39,42,43,44]. Therefore, in order to prevent future cardiovascular events, identification of subjects with masked hypertension or white-coat hypertension is essential to adequately treat masked hypertension and to provide long-term follow-up of white-coat hypertension using home BP measurement.

Then, we started the observational study in 2005 to investigate the effect of proper home BP control on cardiovascular events and mortality among essential hypertensive patients receiving antihypertensive treatment (the J-HOME-Morning study and the J-HOME-Elderly study). The advantages of home BP measurements in the management of treated hypertensive patients have been reported in many other studies [45,46,47]. However, the target BP levels for home BP values have not yet been established. Therefore, we also started the Hypertension Objective treatment based on Measurement by Electrical Devices of Blood Pressure (HOMED-BP) study, which is a large-scale intervention trial to determine both optimal target BP based on home BP and optimal initial antihypertensive medication [48]. It is expected that the management of hypertension based on home BP measurements will be established and spread widely after the establishment of target BP levels based on home BP values.
